# An *In Vitro* Model of the Horse Gut Microbiome Enables Identification of Lactate-Utilizing Bacteria That Differentially Respond to Starch Induction

**DOI:** 10.1371/journal.pone.0077599

**Published:** 2013-10-01

**Authors:** Amy S. Biddle, Samuel J. Black, Jeffrey L. Blanchard

**Affiliations:** 1 Department of Microbiology, University of Massachusetts, Amherst, Massachusetts, United States of America; 2 Department of Veterinary and Animal Science, University of Massachusetts, Amherst, Massachusetts, United States of America; 3 Graduate Program in Molecular and Cellular Biology, University of Massachusetts, Amherst, Massachusetts, United States of America; 4 Department of Biology, University of Massachusetts, Amherst, Massachusetts, United States of America; 5 Graduate Program in Organismal and Evolutionary Biology, University of Massachusetts, Amherst, Massachusetts, United States of America; Charité-University Medicine Berlin, Germany

## Abstract

Laminitis is a chronic, crippling disease triggered by the sudden influx of dietary starch. Starch reaches the hindgut resulting in enrichment of lactic acid bacteria, lactate accumulation, and acidification of the gut contents. Bacterial products enter the bloodstream and precipitate systemic inflammation. Hindgut lactate levels are normally low because specific bacterial groups convert lactate to short chain fatty acids. Why this mechanism fails when lactate levels rapidly rise, and why some hindgut communities can recover is unknown. Fecal samples from three adult horses eating identical diets provided bacterial communities for this *in vitro* study. Triplicate microcosms of fecal slurries were enriched with lactate and/or starch. Metabolic products (short chain fatty acids, headspace gases, and hydrogen sulfide) were measured and microbial community compositions determined using Illumina 16S rRNA sequencing over 12-hour intervals. We report that patterns of change in short chain fatty acid levels and pH in our *in vitro* system are similar to those seen in *in vivo* laminitis induction models. Community differences between microcosms with disparate abilities to clear excess lactate suggest profiles conferring resistance of starch-induction conditions. Where lactate levels recover following starch induction conditions, propionate and acetate levels rise correspondingly and taxa related to *Megasphaera* *elsdenii* reach levels exceeding 70% relative abundance. In lactate and control cultures, taxa related to *Veillonella* *montpellierensis* are enriched as lactate levels fall. Understanding these community differences and factors promoting the growth of specific lactate utilizing taxa may be useful to prevent acidosis under starch-induction conditions.

## Introduction

Horses are hindgut fermenters, adapted to grazing continually on marginal forages that change seasonally, thus slowly [1]. The hindgut (caecum and colon) comprises roughly two thirds of the volume of the equine digestive tract [2]. Here complex plant material is fermented by microbes to short chain fatty acids (SCFA) such as acetate, propionate, and butyrate, which provide 60-70% of the daily energy needs of the horse [3,4]. Rapid dietary change and modern feeding practices of 2-3 meals a day of starch-based concentrate and/or fructans from rich pasture can disrupt normal fermentation in the hindgut, causing lactic acidosis, and colic [5–8], and predisposing animals to bouts of laminitis [9–11].

Laminitis is a chronic, crippling disease, accounting for 15% of all lameness in horses in the United States, with over 27% unable to return to normal work, and 4.7% mortality [12]. It is characterized by weakened adhesion and eventual detachment of the distal phalynx from the lamellae of the inner hoof wall resulting in permanent rotation of the coffin bone and severe pain. Factors released into the bloodstream by bacteria in the gut during lactic acidosis are thought to serve as triggers for dietary laminitis [13-15], however the molecular mechanisms underlying induction are unknown.

Surveys of equine hindgut bacteria using culture based methods [16] and 16S rRNA gene sequencing [17-19] have detected a diverse community of novel microbes dominated by Firmicutes, with Bacteroidetes, Proteobacteria, and Verrucomicrobia as other major phyla. Studies have detected a greater proportion of fibrolytic bacteria than starch and lactate utilizing bacteria in the cecum than in the colon [20,21], reflecting a substrate content normally low in starch and soluble carbohydrates due to the action of endogenous enzymes and absorption of nutrients in the small intestine.

Experimental *in vivo* models of laminitis induction using starch gruel or oligosaccharide [22] have revealed changes in hindgut microbiota during the developmental stage (24-36 hours post induction), correlated with a drop in pH and an increase in lactic acid concentration [23,24]. Lactic acid bacteria, specifically members of the *Streptococcus bovis/ equinus* group (now renamed *Streptococcus* *lutetiensis* [25]), have been implicated as major producers of lactic acid, rapidly increasing in numbers as lactic acid levels rise and the pH drops. At the lowest caecal pH (4-4.5) and levels of lactic acid reaching 1000 µmol/g caecal fluid, acid sensitive fibrolytic and gram negative bacteria die off, while *Lactobacilli sp*. and *Mitzuokella* *sp.* increase [23,26,27]. By 32-36 hr, hindgut lactate levels and pH approach normal levels in most horses [23,28]. In other experiments of starch induction, blood D-lactate levels peaked at 20-24 hr, then declined and disappeared by 36-40 hr [29].

Lactate levels in the hindgut are normally low due to the activity of lactate utilizing bacteria. It is unclear why this mechanism fails during conditions of starch induction. While studies of lactate producers have pointed to specific taxa that proliferate during the developmental stage of laminitis [23,26,27], little is known about how the abundance of lactate utilizing bacteria changes over the same time course, which lactate utilizers survive the drop in pH, and which lactate utilizers are active in the later stages to bring lactate concentrations back to normal levels.

In this study we used fecal samples collected from 3 healthy, adult horses eating an identical pasture based diet in an *in vitro* model system to track bacterial metabolites and community shifts over time in response to enrichment with starch and/or lactate. Patterns of changes in lactate concentration and pH were similar to those reported in published *in vivo* studies [5,23]. Illumina 16s rRNA amplicon sequencing was used to track changes in hindgut microbiota over the time course, specifically identifying lactate utilizing taxa and bacterial groups that proliferated as lactate levels drop. Additionally, we identified community differences in cultures lacking the ability to clear excess lactate, which may lead to further insight into why some horses are resistant to starch induction, and point to bacteria with the potential to attenuate or prevent lactic acidosis.

## Materials and Methods

### Ethics Statement

This study was carried out in accordance with the guidelines set forth by the Morris Animal Foundation and applied by the Institutional Animal Care and Use Committee at the University of Massachusetts, Amherst. We thank Dr. Carlos Gradil (Department of Veterinary and Animal Sciences, University of Massachusetts, Amherst, MA) for generously lending his time and expertise in sampling, and the Hadley Farm, University of Massachusetts, Amherst, MA for providing three horses for this study.

### Sampling and in vitro enrichments

Fecal samples were manually collected from the midrectum of three Morgan geldings cohoused at the University of Massachusetts, Hadley Farm and fed identical hay based diets. None of the animals had received antibiotics, anthelmenthics, or other medications for at least three months prior to sampling. Samples were protected from oxygen exposure during collection by inverting the glove around each as it was removed, and placing each immediately in a container evacuated of oxygen. Samples were kept as close to 39°C as possible in a heated, insulated container during transport and the following steps of dilution and inoculation. In an anaerobic chamber, samples were diluted to make a 10% slurry in anaerobic dilution media prepared as described by Bryant [30]. This slurry was used to inoculate basal media described by de Carvalho [31] at 2.5% which was enriched with either 1% soluble starch, 50 mM sodium lactate, or both 1% soluble starch and 50 mM sodium lactate. Control cultures were diluted at 2.5% of basal media with no enrichment. Cultures were kept under anaerobic conditions, incubated at 39°C and sampled every 12 hours for metabolite analysis and DNA extraction. Sampling and enrichments were subsequently repeated for the same horses, inoculant concentrations, culture and incubation conditions in 125 ml serum bottles (Wheaton, Millville, NJ) fitted with stoppers that enable headspace gas collection and analysis.

### Metabolite measurements

Short chain fatty acids (acetate, lactate, butyrate, succinate, formate, and propionate) were measured for samples taken over the time course using high performance liquid chromatography (HPLC) (Shimadzu, Japan) with an Aminex HPX-87H column (Bio-Rad, Hercules, CA). Headspace gases were analyzed using a gas chromatograph (Shimadzu GC-8A, Shimadzu, Japan) fitted with a HayeSep DB column 100/120 (Bandera, TX). Hydrogen sulfide levels were measured using the methylene blue assay as in Cline [32] modified for culture samples as follows: at each time point, 1.0 ml of culture was removed from each serum bottle via syringe, transferred into sealed vials containing an equal volume of 1.2% degassed zinc acetate solution, and stored at 4°C until all samples were collected. To a 1.0 ml subsample, 62.5 µl of 7% sodium hydroxide was added. Following a 15 min incubation at room temperature, 187.5 µl of 0.1% N,N’-dimethyl-*p*-phenylenediamine and 187.5 µl 10 mM iron (III) chloride were added, stoppered immediately, and incubated for 20 minutes at room temperature. The resulting suspension was spun down and the absorbance of the supernatant was measured at 670 nm in comparison to standard solutions (0-.55mM sodium sulfide, and uninoculated media controls). pH was determined using EMD colorphast pH strips (Fischer Scientific, Pittsburgh, PA).

### DNA extraction, amplification, and sequencing

DNA was extracted from each sample as described elsewhere [33,34] with the following modifications. Samples were subjected to two extractions in Matrix E bead tubes containing 5% CTAB in 1 M NaCl and .25M phosphate buffer (pH 8), phenol: chloroform: isopropyl alcohol (25:24:1), and 0.1 M ammonium aluminium sulfate, followed by separation with 24:1 chloroform: isoamyl alcohol. DNA was then precipitated with 30% polyethylene glycol 6000 in 1.6 M NaCl, washed with 70% ethanol, and resuspended in 10 mM Tris, pH 8. Replicate extractions were pooled and purified using MoBio PowerClean DNA clean-up kit (MoBio, Carlsbad, Ca) and quantified using Quant-iT PicoGreen assay (Invitrogen, Carlsbad, Ca).

Amplification of the V4 region of the 16S rRNA gene and attachment of Illumina adaptors and bar codes for multiplexing samples (reverse read only) was done in triplicate as described elsewhere [35]. Briefly, genomic DNA was amplified with universal forward 515F (5’- Illumina adapter- Forward primer pad- Forward primer linker- GTGCCAGCMGCCGCGGTAA-3’) and universal reverse 806R (5’- Illumina adapter- Golay bar code- Reverse primer pad- Reverse primer linker- GGACTACHVGGGTWTCTAAT-3’). Each PCR reaction mixture contained 10 ng of genomic DNA, 2.5 µl 10X buffer, 2.0 µl MgCl_2_ (25 mM), 2.0 µl dNTP (2.5 mM each), 5.0 mM (each) forward and reverse primers, 1.25 µl (25 µg) BSA (Roche, Indianapolis, IN), 0.25 µl (1.25 U) *Ex Taq* (TaKaRa, Japan), and molecular grade water to reach a volume of 25 µl. PCR was performed with 3 min of initial denaturation at 94°C followed by 30 cycles of the following program (denaturation, 94 °C for 45 sec; annealing, 50 °C for 30 sec; and extension, 72 °C for 45 sec) followed by a final extension at 72 °C for 7 min. PCR products were cleaned using Qiagen MinElute kit (Qiagen, Valencia, Ca) as directed and quantified using the Quant-iT PicoGreen assay (Invitrogen, Carlsbad, Ca). Equal concentrations were pooled and sequenced using the Illumina MiSeq platform at the Dana Farber Cancer Institute, Molecular Biology Core Facilities (Cambridge, MA).

### Sequence analysis

Sequences were demultiplexed and trimmed of bar codes and primer sequences, then filtered for quality and reverse and forward reads were assembled into contigs using FastQC [36]. Sequence processing was done in QIIME [37] using the following workflow: Reads were aligned using default parameters (PyNAST) [38], operational taxonomic units (otus) were picked at the 97% similarity threshold using the subsampled open-reference option, chimeric sequences were identified using ChimeraSlayer [39] and removed, taxonomic assignments were made against the most recent greengenes database (October, 2012) [40]. Sequence data has been submitted to the NCBI Sequence Read Archive (SRA), Accession number: SRP028582.

## Results

### Patterns of changes in metabolites

**Figure 1:**
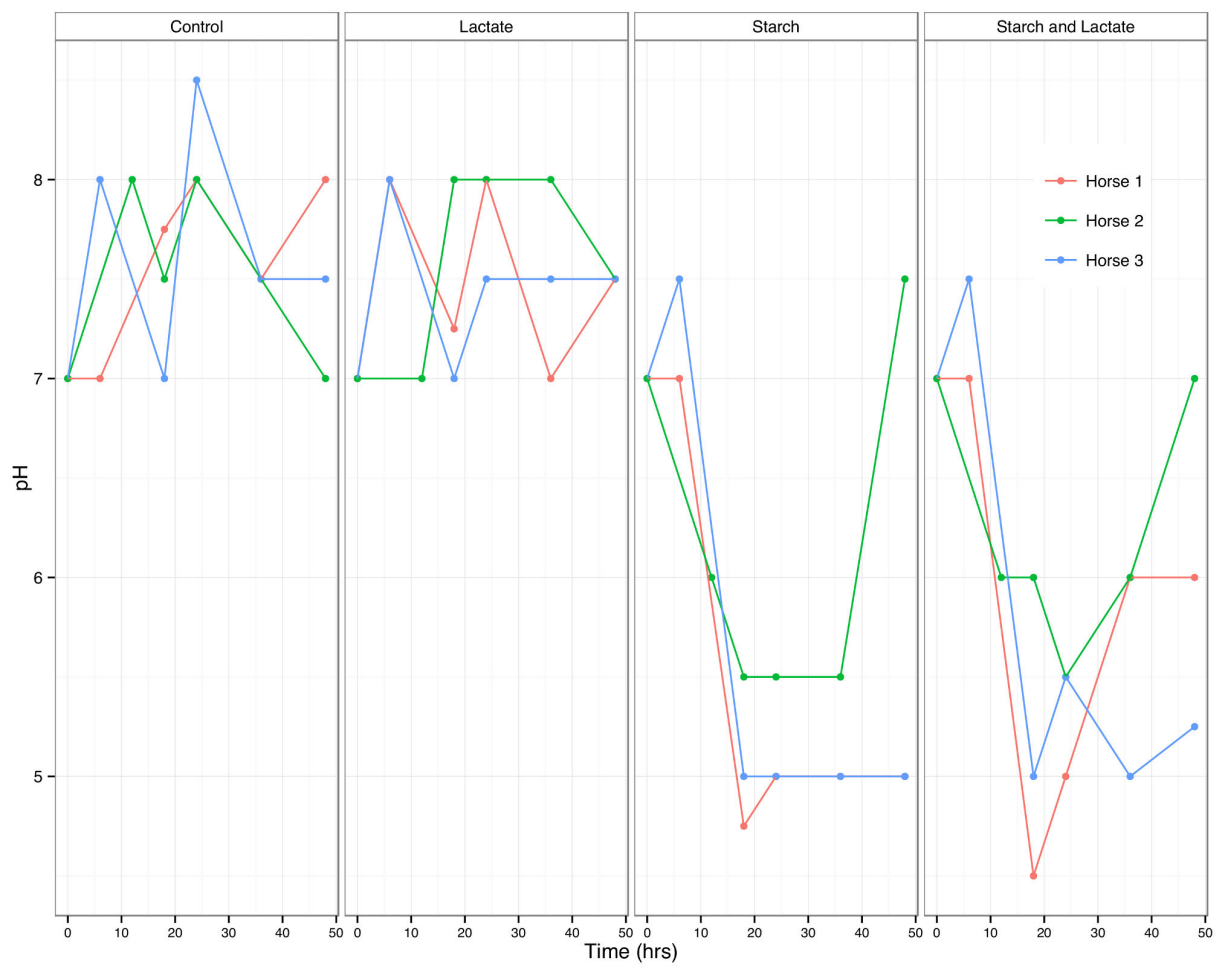
pH of cultures over time by horse. pH of cultures from each horse and culture condition measured at 6 hour intervals.

**Figure 2:**
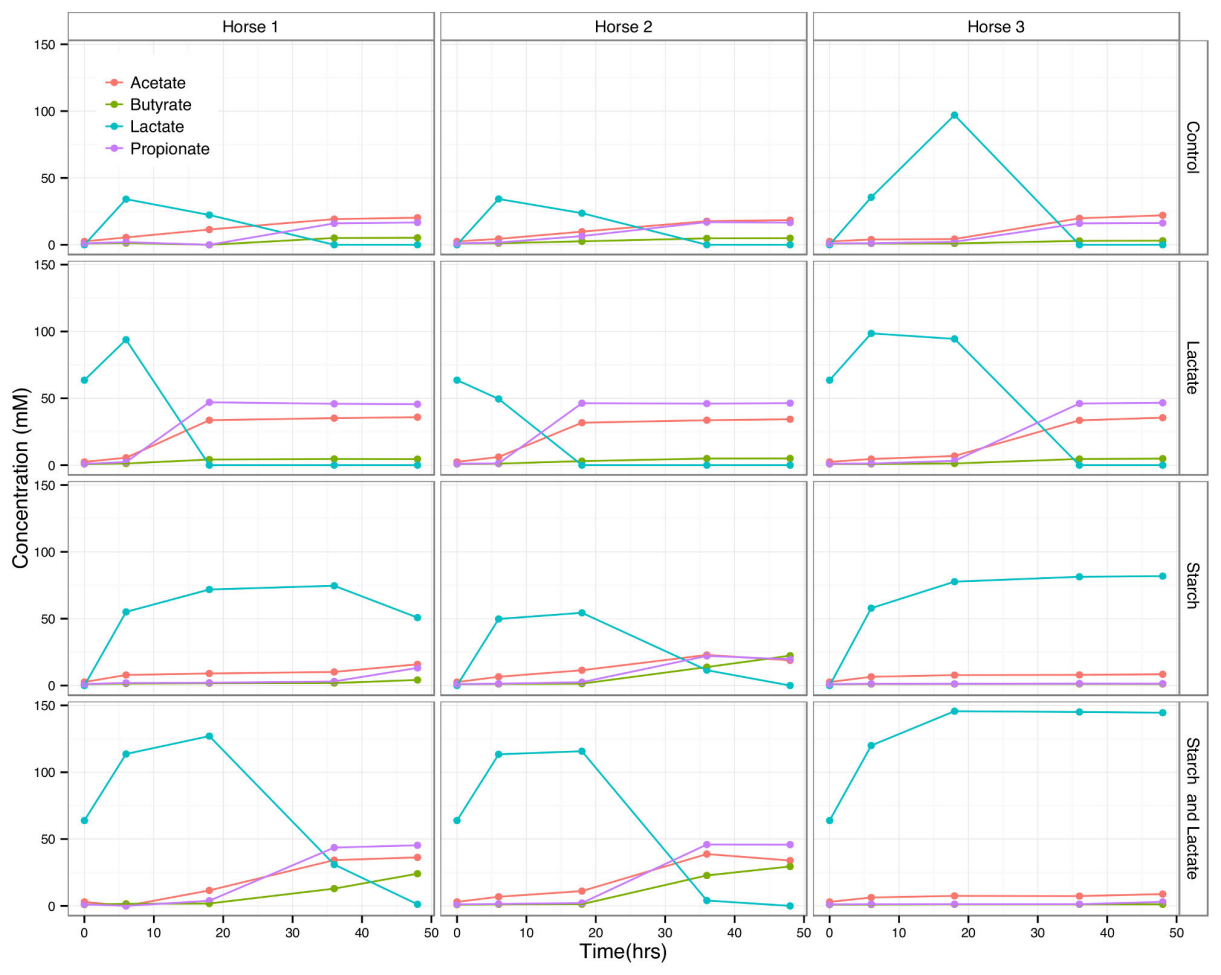
Short chain fatty acid metabolites over time by horse. Concentration (mM) of acetate, butyrate, lactate, and propionate measured by high performance liquid chromatography from each horse and culture condition at times 0, 6, 18, 36, and 48 hours.

Despite variation in the pH response between the three horses, in all of the starch and starch/lactate enrichments, the pH dropped to below 6 by hour 12, and reached levels between 4 and 5 by hour 18 (Figure 1). These values paralleled the peak in lactate levels for these cultures over the same time course (Figure 2). The control and lactate enriched treatment groups showed an initial increase in lactate followed by a rapid decline as the lactate was used up. In the starch enriched cultures, lactate levels peaked by hour 18, exceeding 100 mM in cultures enriched with both starch and lactate. Where lactate levels dropped over time, there was a corresponding increase in acetate, propionate, and butyrate.

The three horse cultures differed dramatically in the ability to attenuate accumulated lactate. The lactate was reduced to below detectable limits in the control and lactate enriched treatment groups of all three horse cultures, however the peak was higher for horse 3 cultures and took longer to drop. In the starch and starch/lactate enrichments the differences were more dramatic. Lactate persisted at maximum levels in horse 3 cultures over the full time course while dropping for horse 1 and 2 cultures by hour 36.

Headspace gases (hydrogen and methane) and hydrogen sulfide levels measured at 12 hour intervals did not show consistent differences between treatment and control conditions due to variation between horses especially for the starch and starch/lactate cultures (Figure S1 and Figure S2).

### Sequence metrics

As shown in Table 1, there were over 6 million sequences and 41,000 OTUs in the dataset following quality filtering and initial OTU picking in QIIME [37]. Chimera removal using Chimera Slayer [39] left more than 2.5 million sequences and 32,000 OTUs for further analysis. Average read length was 254 bp.

**Table 1:** Sequence metrics.

	Before Chimera Check	After Chimera Check
Total number of sequences	6,043,194	2,576,535
Sequences per sample (mean)	125,900	53,678
Sequences per sample (min)	51,890	9,685
Number of OTUs	41,055	32,563

Total number of sequences, sequences per sample, and OTUs before and after chimera detection using Chimera Slayer.

Rarefaction curves of the number of sequences per sample by observed species (Figure S3) indicated a leveling off in terms of new species at the minimum sequence depth of 9685. Since 83% of the samples had over twice this depth, and Good’s coverage at the depth of the smallest library (9685) was 89% (Table S1), we are confident that our sequencing efforts have captured most of the diversity in these samples.

### Relative abundance at the phylum level

**Figure 3:**
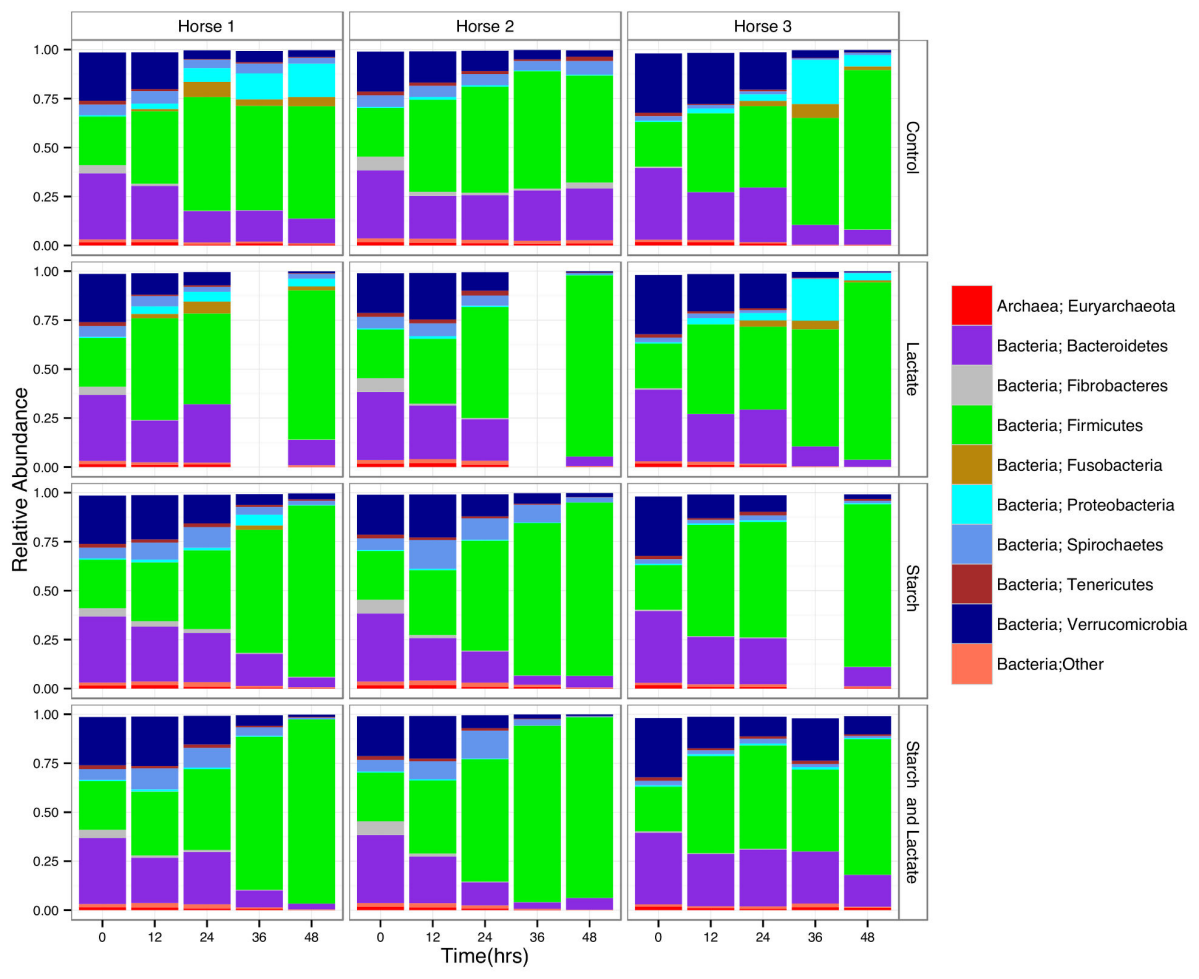
Distribution of sequences by phyla. Relative abundance of sequences for each operational taxonomic unit found at or greater than 1% at each time point for each culture condition and horse sample by phyla.

Taxonomic assignments at the phylum level (Figure 3) showed that the Firmicutes were the most abundant group by time 48 in all cultures regardless of enrichment conditions, with a corresponding decline in all other major groups, namely the Verrucomicrobia, Spirochaetes, Proteobacteria, and Bacteroidetes, however at this taxonomic level, clear differences between horse and treatment groups were not apparent.

### Relative abundance at the family level for Firmicutes

**Figure 4:**
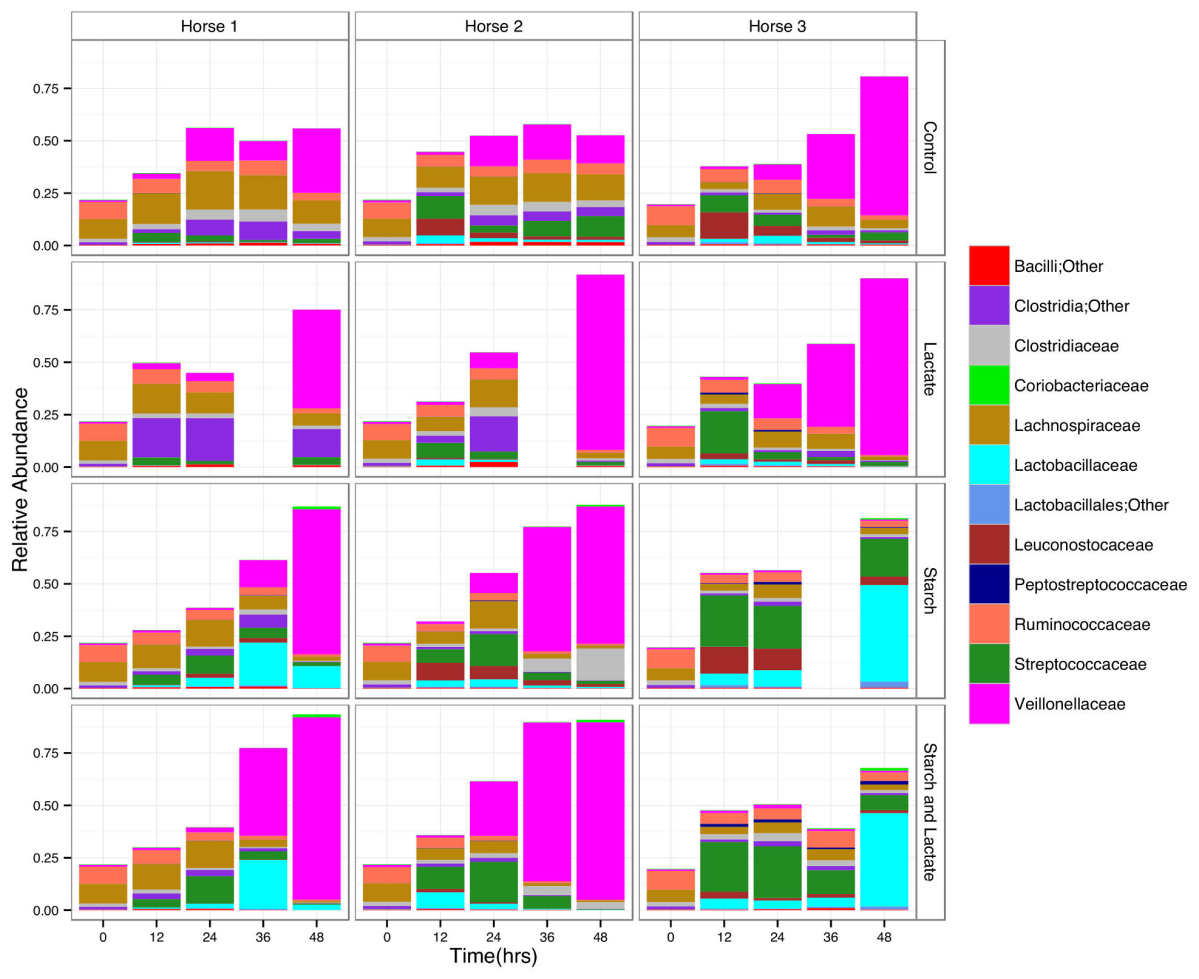
Distribution of Firmicute sequences by family. Relative abundance of Firmicutes sequences found at or greater than 1% by family at each time point for each culture condition and horse sample.

Differences between horse cultures and treatment conditions were evident at the family level, specifically for the most abundant phyla, the Firmicutes. The Veillonellaceae, a family with known lactate utilizing species [41,42], increased in abundance in all cultures in the control and lactate treatment groups. In the starch and starch/lactate enrichments there were dramatic differences between horse 3 cultures and those of horse 1 and 2 (Figure 4). By time 48, the Veillonellaceae dropped to less than 5% in horse 3 cultures, while in horse 1 and 2 cultures this group was the highest in abundance, making up more than 70% of the total sequences (Figure 4). The most abundant family for the starch and starch/lactate horse 3 cultures at time 48 were the Lactobacillaceae, making up greater than 40% of the total sequences at this time point. The Streptococcaceae reached a peak in abundance by 24 hours in the starch and starch/ lactate conditions for all horse cultures, after which the abundance dropped. In horse 1 and 3 cultures this decrease was accompanied by an increase in the Lactobacillaceae, however in horse 2 the abundance of Lactobacillaceae remained relatively low even as the Streptococcaceae declined over time.

### Distribution of abundant OTUs

**Figure 5:**
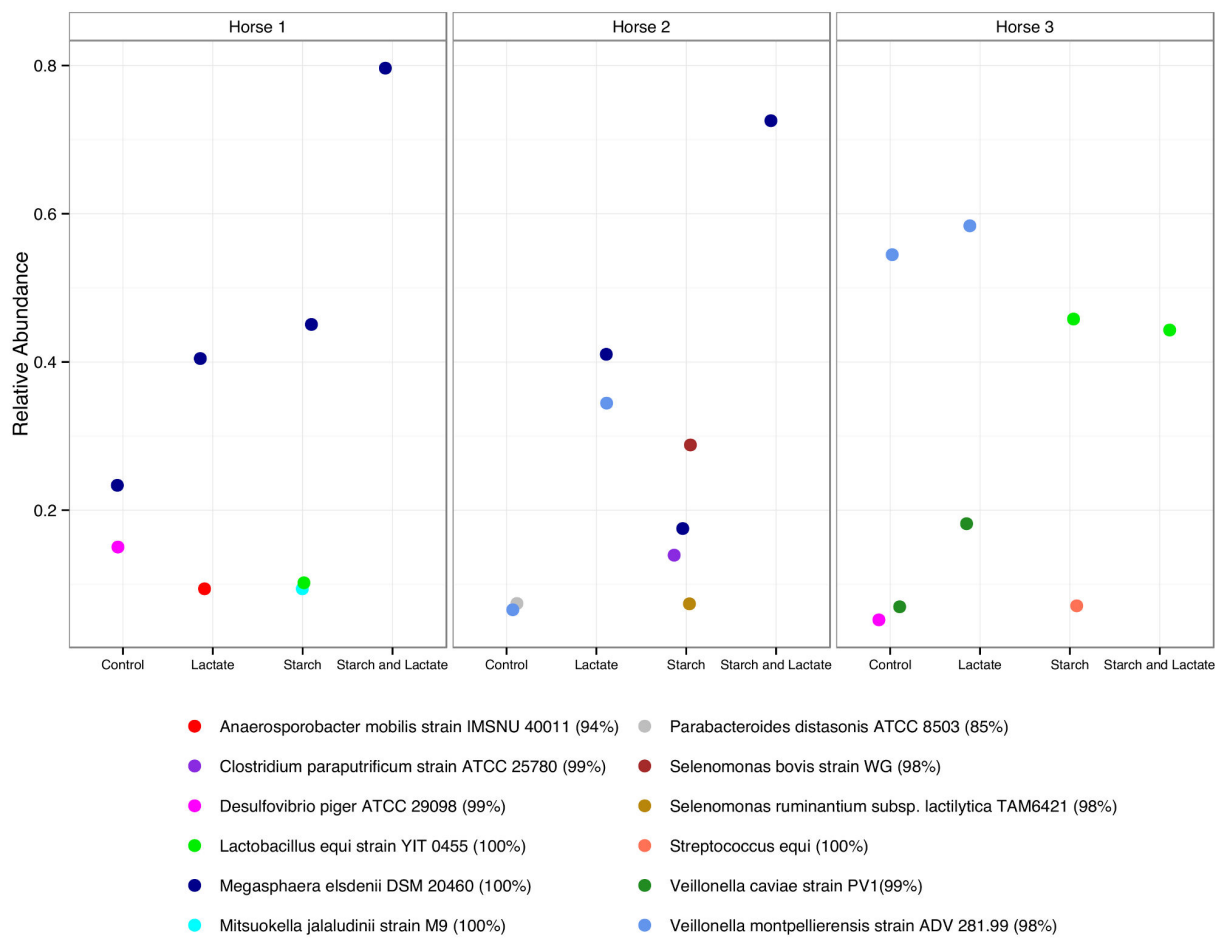
Distribution of most abundant OTUs at time 48. Relative abundance of OTUs found at greater than 5% in each culture condition by horse. OTUs are identified by best BLAST match, and percent sequence similarity is given.

Identification of specific taxa that were most abundant by hour 48 (Figure 5 and Table 2) suggested community differences between horse cultures of specific interest in light of differences in lactate utilization and attentuation. There was a striking difference in OTU abundance and distribution between horse 3 cultures and those of horse 1 and 2 in all treatment conditions at hour 48. While the most abundant sequence (between 50-60% relative abundance, with 98% identity to *Veillonella* *montpellierensis* ) under control and lactate conditions for horse 3 was also found in horse 1 and 2 cultures, it reached a lower abundance in both. While horse 3 control and lactate enriched cultures were able to attenuate lactate, it persisted in high concentrations in starch and starch/lactate enrichments in which no member of the Veillonellaceae was highly abundant.

**Table 2:** Best BLAST hits for most abundant OTUs at Time 48.

Best BLAST hit	Percent similarity	NCBI Accession Number
Parabacteroides distasonis ATCC 8503	85%	NR_074376.1
Streptococcus equi subsp. zooepidemicus MGCS10565	100%	NR_102812.1
Desulfovibrio piger ATCC 29098	99%	NR_041778.1
Clostridium paraputrificum ATCC 25780	99%	NR_026135.1
Mitsuokella jalaludinii M9	100%	NR_028840.1
Megasphaera elsdenii DSM 20460	100%	NR_102980.1
Anaerosporobacter mobilis IMSNU 40011	94%	NR_042953.1
Selenomonas bovis strain WG	98%	NR_044111.1
Veillonella montpellierensis ADV 281.99	98%	NR_028839.1
Lactobacillus equi YIT 0455	100%	NR_028623.1
Selenomonas ruminantium subsp. lactilytica TAM6421	98%	NR_075026.1
Veillonella caviae PV1	99%	NR_025762.1

Best BLAST it from the 16S rRNA Reference Sequence Database, Percent similarity and NCBI accession numbers for the reference sequence

**Figure 6:**
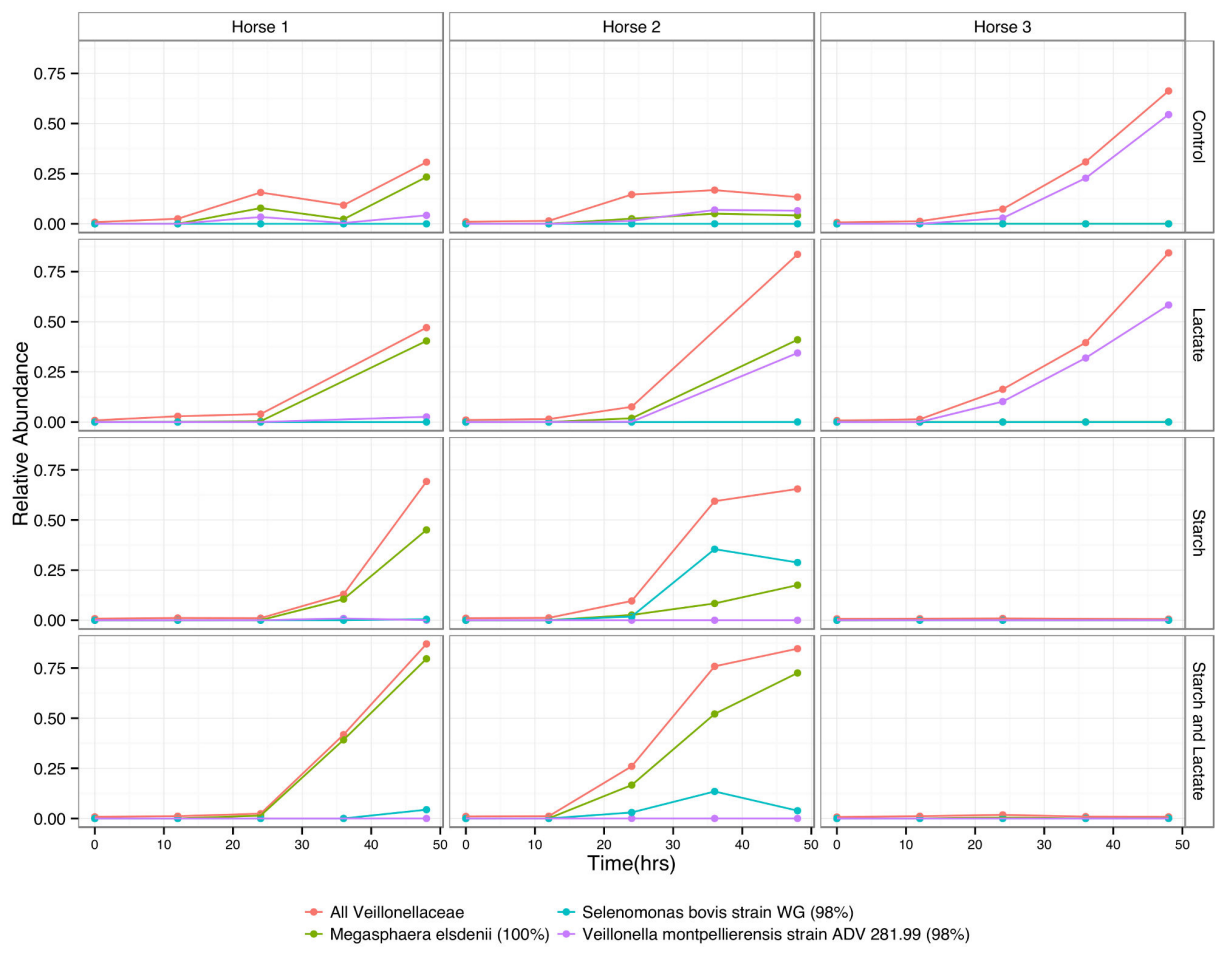
Change in most abundant Veillonellaceae OTUs over time. Relative abundance of most abundant Veillonellaceae OTUs over time in each culture condition by horse. OTUs are identified by best BLAST match, and percent sequence similarity is given.

A different OTU, related to *Megasphaera* *elsdenii* (100% identity) was the most abundant OTU in horse 1 and 2 cultures, especially in the starch and starch and lactate enrichments, reaching relative abundances of over 70%. While this OTU was present in single numbers at Time 0 in all three horse samples, and persisted in horse 3 in low numbers under all culture conditions (Figure 6), it never reached abundances of greater than 0.40% at any condition or time point.

**Figure 7:**
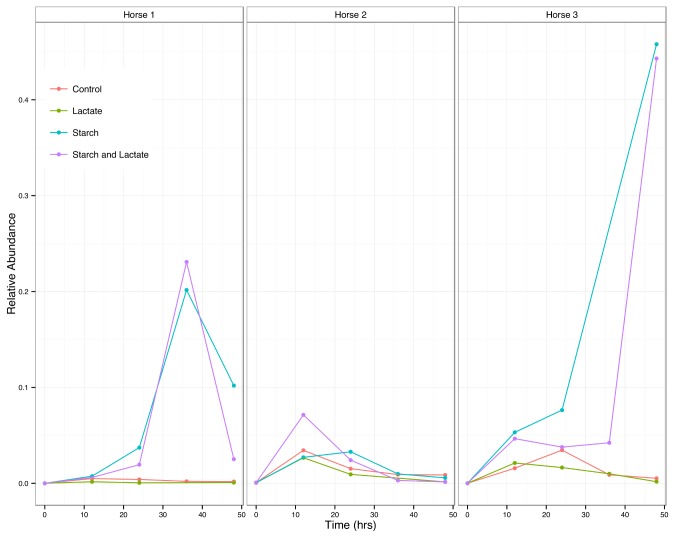
Change in single, abundant Lactobacillaceae OTU over time. Relative abundance of the single, dominant Lactobacillus OTU over time for each culture condition by horse.

The most abundant OTU in horse 3 starch and starch and lactate cultures was related to *Lactobacillus* *equi* (100% sequence identity) and represented 97.7% of the Lactobacillaceae sequences across the dataset. This OTU was present in all three horse cultures, reaching abundances of 20% and greater in horse 1 in starch and starch and lactate enrichments at time 36, but then falling to 10% or less by time 48 (Figure 7).

**Figure 8:**
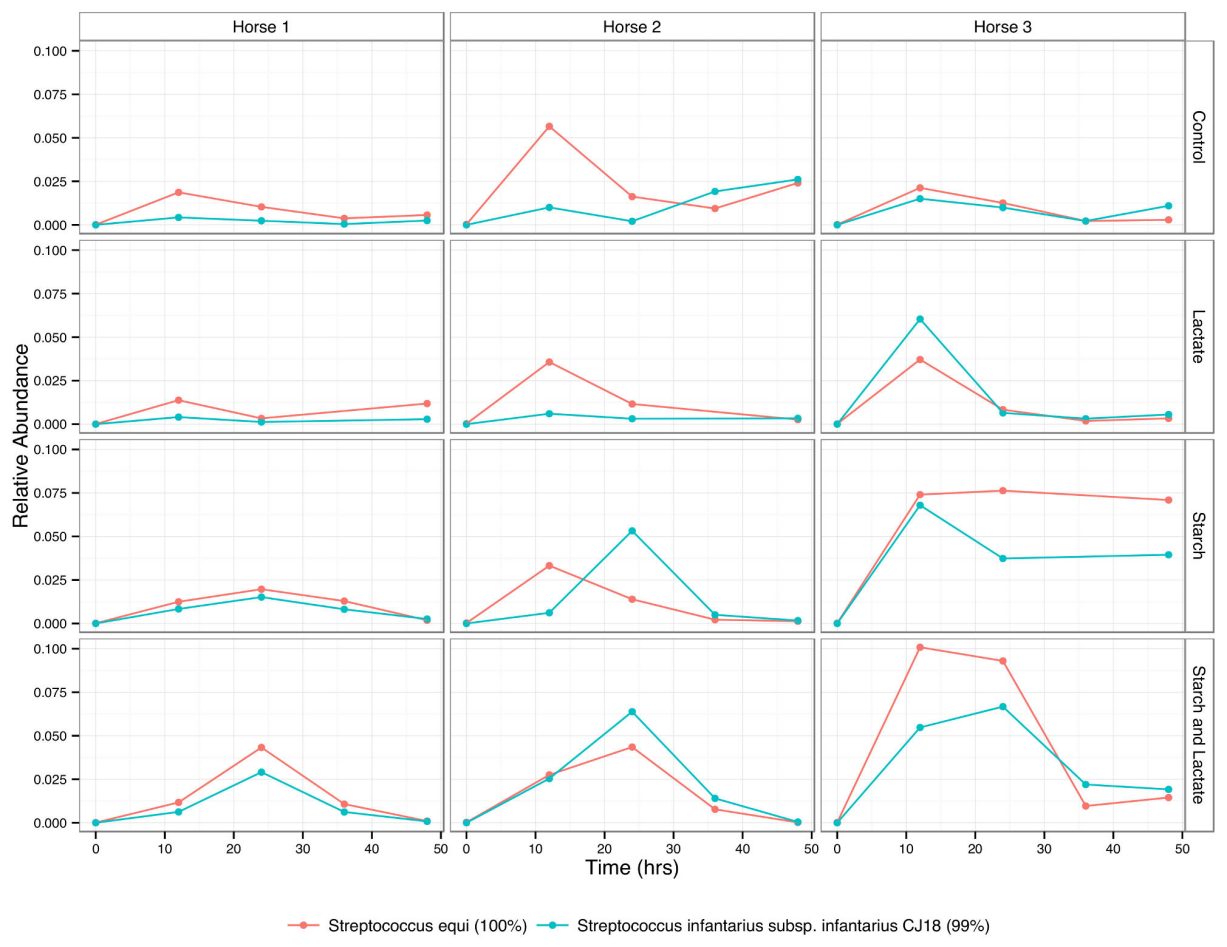
Change in abundant Streptococcaceae OTUs over time. Relative abundance of dominant Streptococcus OTUs over time for each culture condition by horse. OTUs are identified by best BLAST match, and percent sequence similarity is given.

Instead of being dominated by one or two abundant OTUs, the distribution of the Streptococcaceae taxa was more dispersed, with the two most abundant OTUs (related to *Streptococcus equi* and *Streptococcus* *infantarius* with 100% and 99% sequence identities respectively) together accounting for less than 50% of the sequences in this group (Figure 8).

## Discussion

### Our in vitro system captures elements observed in vivo

The accumulation of lactate has long been recognized as an early event in the microbial response to carbohydrate overload leading to colic and laminitis in horses [5,10]. In the *in vitro* system described herein, we were able to simulate many key aspects of starch induced conditions that have been reported elsewhere for *in vivo* experiments. The extent and timing of pH changes (Figure 1) and fluctuations of SCFA levels (Figure 2), especially with respect to lactate, acetate, and propionate are similar to those reported for *in vivo* starch induction studies [5,23].

No study thus far has tracked microbial changes over the time course of starch induction in horses using 16S rRNA deep sequencing as described here, however, the changes that we observed in community composition are similar to what has been reported in equine and ruminant culture and probe based studies [7,14,43,44]. Specifically, in the first 24 hours following enrichment, we noted an increase in Firmicutes, especially members of the Streptococcaceae, coinciding with a decrease in fibrolytic groups (Ruminococcaceae and Lachnospiraceae), followed by an increase in the Lactobacillaceae as the abundance of Streptococcaceae falls.

Despite a sample size of three horses, we were able to observe between-horse differences in the ability to attenuate accumulated lactate as has been described in previous studies [5,23].

Certainly the microbial dynamics reported here may not reflect the actual community compositions of the caecum and large intestine of horses who recover or resist conditions of lactic acidosis, however the elucidation of endogenous lactate utilizers that thrive under conditions of low pH following starch enrichment in our *in vitro* model may provide insight into microbial mechanisms of resistance and/or recovery.

### Lactate producing bacteria proliferate following starch induction

While we observed the pattern of increase in *Streptococcus* species during the first 24 hours of starch induction followed by an increase in *Lactobacilli* species noted in other studies [7,23] we expected the *Streptococcus* *lutetiensis* group (formerly *Streptococcus bovis* [25]) to be more highly represented in the starch and starch/lactate cultures, as it has been identified as the major lactate producer in *in vivo* starch induction studies [7,24,29]. Our data indicates a high abundance of the family Streptococcaceae in the starch and starch/lactate cultures, but fails to resolve subtle differences between species. We recognize that basing taxonomic assignments on a single region (V4) of the16S gene is inherently limited [45,46] for all bacterial groups since 500-700 bases are recommended for species resolution [47], and suspect that taxa within the *Streptococcus* *lutetiensis* group may be especially difficult to distinguish due to their close phylogenetic relationships [25,48]. Sequencing with longer reads would clarify the species composition of this family in our starch and starch/lactate enrichments.

### Members of the Veillonellaceae are the most abundant lactate utilizing bacteria

While studies have pointed to *Streptococcus* *lutetiensis* and *Lactobacillus* sp as major lactic acid producers [7,28,29], relatively little attention has been paid to bacteria in the horse gut that actively utilize lactic acid. Studies of lactic acidosis in ruminants [43,44,49] have identified specific genera, namely *Megasphaera* , *Veillonella* , *Selenomonas* , *Propionibacterium* , and *Anaerovibrio* as key lactate utilizers. In fact, strains of *Megasphaera* *elsdenii* have been shown to be effective in preventing lactic acidosis in cattle and are under development as probiotic therapies [50–52].

Using deep sequencing of the 16S rRNA gene of fecal communities challenged with starch, lactate, or both in an *in vitro* model of starch induction, we report here changes in the abundance of specific microbes associated with the reduction of lactic acid. As we observed at time 0 in this study, 16S rRNA surveys of the equine gut microbiome have shown that under normal conditions, the Veillonellaceae (known lactate utilizers) comprise 1% or less of the total bacterial abundance [17–19]. One probe-based *in vivo* study did not see a difference in the abundance of Veillonellaceae in response to dietary change despite an increase in lactate levels [24]. It is unclear why the Veillonellaceae in that study did not increase in abundance as lactate accumulated, or which specific taxa were present in those horses.

In our study we observed that one particular taxa closely related to *Megasphaera* *elsdenii* was highly abundant in all starch and starch/lactate cultures in which lactate accumulated and was attenuated. This taxa was present in very low abundance in cultures in which lactate persisted. It is unclear why this taxa fails to proliferate in any of the horse 3 cultures while reaching such high abundances under all conditions in horse 1 and 2 cultures. A second OTU related to *Veillonella* *montpellierensis* was highly abundant in control and lactate enrichments specifically in horse 2 and 3 cultures, but did not thrive in the starch or starch/lactate enrichments, suggesting that other factors such as pH or competitive interactions exert selective pressure under starch and starch/lactate enrichment conditions.

While our data indicates a relationship between the presence of the *Megasphaera* *elsdenii* OTU and the reduction of lactate, conclusive evidence that this taxa is responsible for reducing lactate levels will require further study. It is possible that other community members with lactate utilizing capabilities are playing active roles as well.

Understanding the factors stimulating or preventing the proliferation of lactate utilizers in the horse gut microbiome could provide valuable information about why some horses are more sensitive to starch induction, and the microbial basis behind mechanisms of resistance.

## Conclusions

A robust *in vitro* model for starch induced laminitis in horses as described here could provide a convenient and cost effective means to understand the microbial dynamics underlying colic and laminitis, and test hypotheses for ways to prevent or interrupt the progress of these equine diseases. Specific taxa in the family Veillonellaceae were highly abundant in starch enriched cultures that were able to attenuate lactate. These could provide useful insights into mechanisms of recovery or resistance, and could be valuable, individually or in consortia, as probiotics to prevent starch induced colic and laminitis.

## Supporting Information

Figure S1 **Hydrogen sulfide concentrations over time by horse.** Concentration (mM) of hydrogen sulfide measured by the Cline (methylene blue) assay from each horse and culture condition at 12 hour intervals.
(TIF)

Figure S2 **Headspace gas concentrations over time by horse.** Concentration (mM) of hydrogen and methane gases measured by gas chromatography from each horse and culture condition at times 9, 20, 32, and 45 hours.
(TIF)

Figure S3 **Rarefaction curves by horse.** Observed species by number of sequences per sample for each horse dataset generated using a sampling depth of 9685 (the minimum number of sequences per sample and default parameters in QIIME.)
(TIF)

Table S1 **Alpha diversity estimates for each sample.** Chao1, observed species, Shannon Index and Good’s coverage for each horse dataset generated using default parameters in QIIME.
(XLSX)
