# Supplementary figures and images for: An In Vitro Model of the Horse Gut Microbiome Enables Identification of Lactate-Utilizing Bacteria That Differentially Respond to Starch Induction
Source: PLoS One. 2013 Oct 1;8(10):e77599. doi: 10.1371/journal.pone.0077599 (PMC3788102; doi:10.1371/journal.pone.0077599)

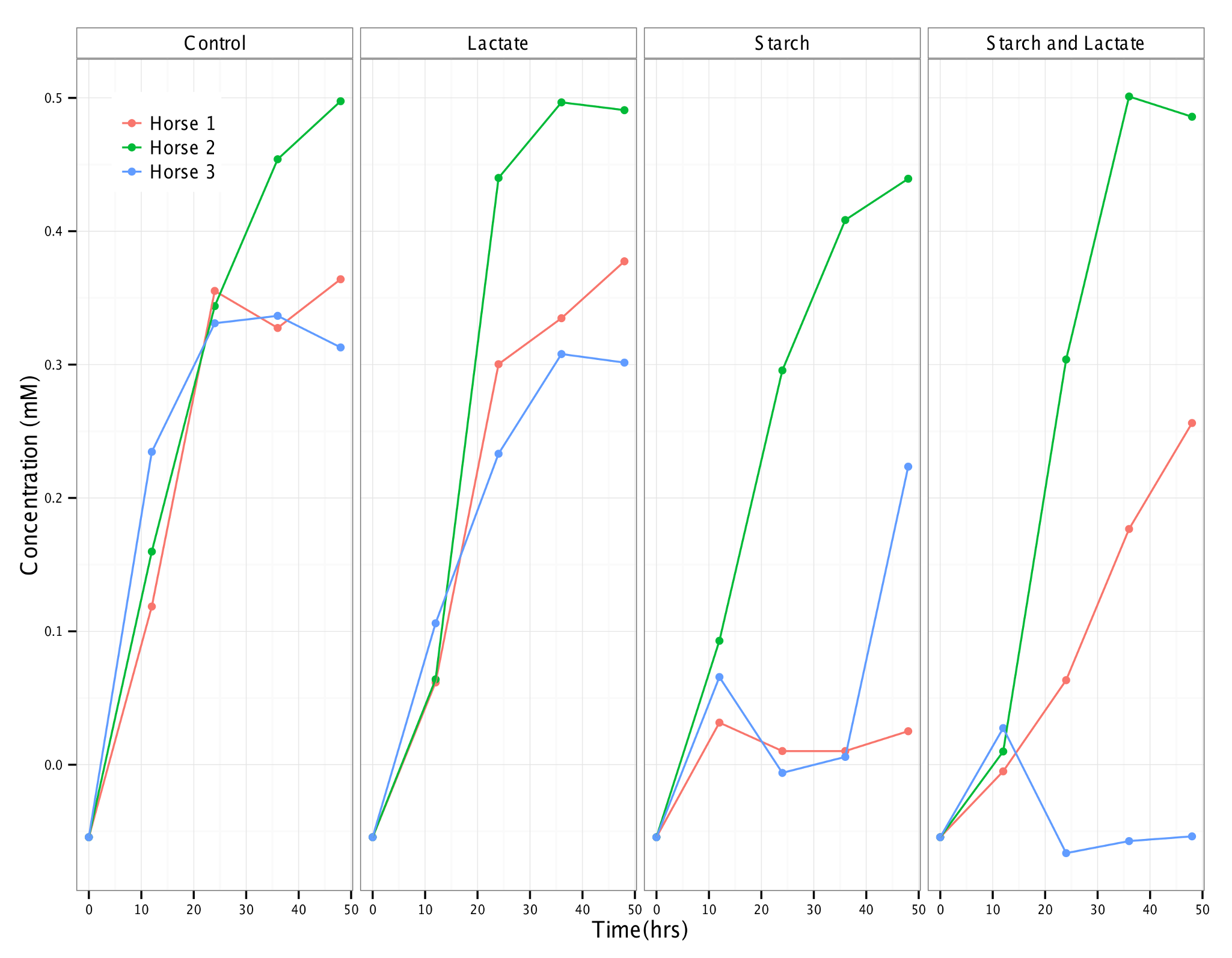

Supplement: Figure S1 — Hydrogen sulfide concentrations over time by horse. Concentration (mM) of hydrogen sulfide measured by the Cline (methylene blue) assay from each horse and culture condition at 12 hour intervals. (TIF) [file pone.0077599.s001.tif]

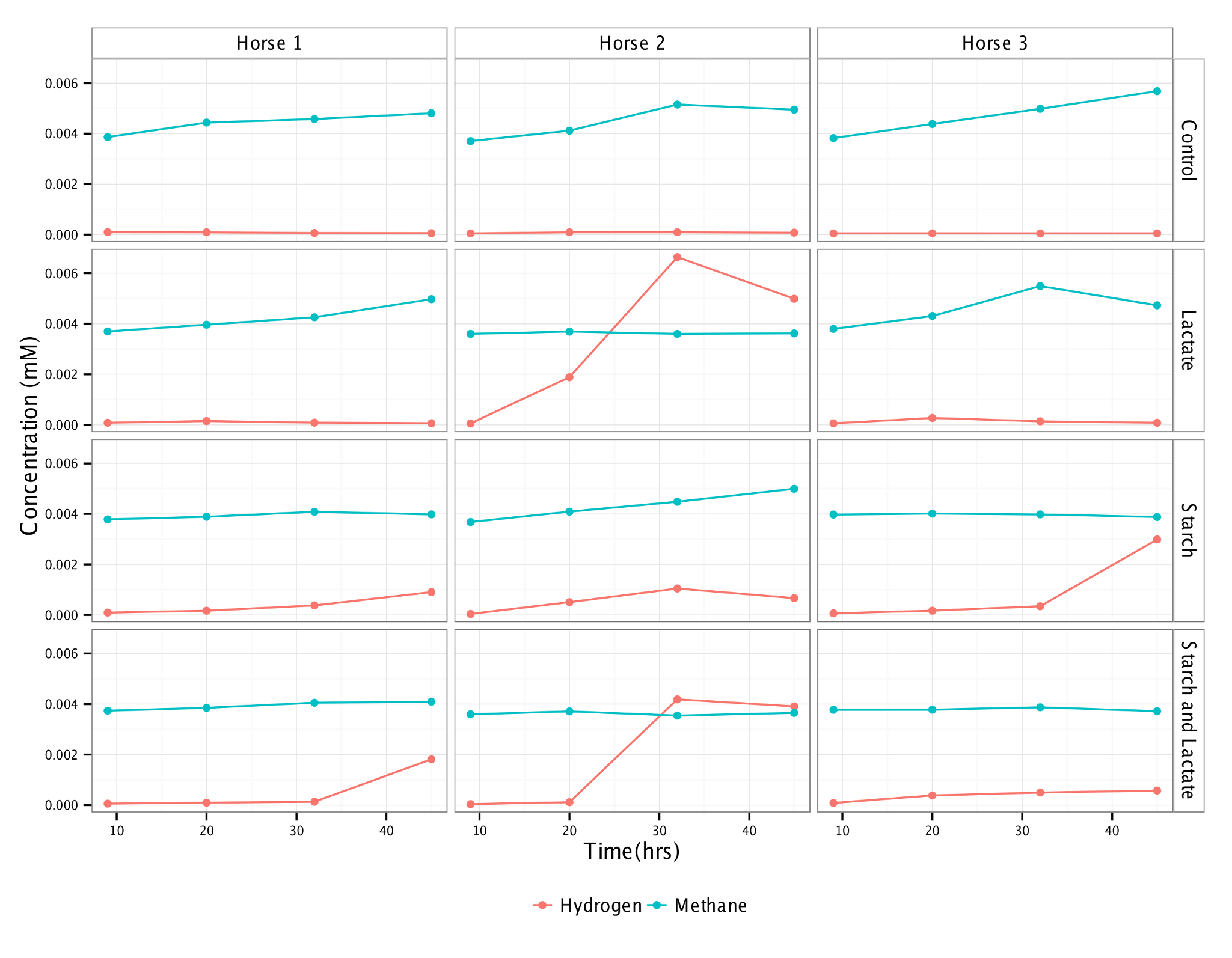

Supplement: Figure S2 — Headspace gas concentrations over time by horse. Concentration (mM) of hydrogen and methane gases measured by gas chromatography from each horse and culture condition at times 9, 20, 32, and 45 hours. (TIF) [file pone.0077599.s002.tif]

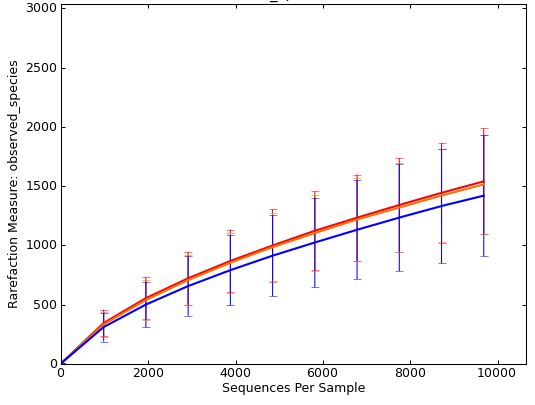

Supplement: Figure S3 — Rarefaction curves by horse. Observed species by number of sequences per sample for each horse dataset generated using a sampling depth of 9685 (the minimum number of sequences per sample and default parameters in QIIME.) (TIF) [file pone.0077599.s003.tif]
